# Metal Zn(II), Cu(II), Ni (II) complexes of ursodeoxycholic acid as putative anticancer agents

**DOI:** 10.1080/13102818.2014.927973

**Published:** 2014-08-30

**Authors:** Lora Dyakova, Daniela-Cristina Culita, Gabriela Marinescu, Marin Alexandrov, Reni Kalfin, Luminita Patron, Radostina Alexandrova

**Affiliations:** ^a^ Institute of Neurobiology, Department of Synaptic Signaling and Communications, Bulgarian Academy of Sciences, Sofia, Bulgaria; ^b^ Institute of Physical Chemistry ‘Ilie Murgulescu’, Romanian Academy, Bucharest, Romania; ^c^ Institute of Experimental Morphology, Pathology and Anthropology with Museum, Department of Pathology, Bulgarian Academy of Sciences, Sofia, Bulgaria

**Keywords:** ursodeoxycholic acid, metal complexes, cytotoxic/cytostatic activity, cell lines, pharmaceutical biotechnology

## Abstract

The aim of the study was to evaluate the influence of metal [Zn(II), Cu(II), Ni(II)] complexes with ursodeoxycholic acid (UDCA) on the viability and proliferation of tumour and non-tumour cells. Cell lines established from retrovirus-transformed chicken hepatoma (LSCC-SF-Mc29) and rat sarcoma (LSR-SF-SR) as well as from human cancers of the breast (MCF-7), uterine cervix (HeLa), lung (A549) and liver (HepG2) were used as model systems. Non-tumour human embryo (Lep-3) cells were also included in some of the experiments. The investigations were carried out by the thiazolyl blue tetrazolium bromide (MTT) test, neutral red uptake cytotoxicity assay, crystal violet staining, double staining with acridine orange and propidium iodide and the colony-forming method. The results obtained revealed that: (1) UDCA and its metal complexes in the tested concentrations decreased (to a varying degree) the viability and proliferation of the treated cells in a time- and concentration-dependent manner; (2) chicken hepatoma (LSCC-SF-Mc29) cells were most sensitive to the cytotoxic and antiproliferative action of the compounds tested, followed by rat sarcoma (LSR-SF-SR) cells; (3) Cu‒UDCA and Ni‒UDCA were more effective against animal LSCC-SF-Mc29 and LSR-SF-SR cells, while Zn‒UDCA significantly decreased the viability and proliferation of human tumour cell lines; (4) applied independently, UDCA expressed lower cytotoxic/cytostatic activity as compared to metal complexes; and (5) the sensitivity of the non-tumour embryonic Lep-3 cells to the effects of UDCA and its metal complexes was comparable or even higher than those of the human tumour cells.

## Introduction

Bile acids (BAs) are a group of acidic steroids with specific physical, chemical and biological characteristics. Primary BAs (e.g. cholic and chenodeoxycholic acid) are directly synthesized from cholesterol by hepatocytes, by the addition of hydroxyl groups and the oxidation of its side chain to form a more water-soluble end product. Secondary BAs [e.g. deoxycholic, lithocholic, ursodeoxycholic acid (UDCA)] are generated in the intestine by bacterial biotransformation of primary BAs.[1,2]

BAs are involved in cholesterol catabolism and intestinal lipid emulsification as detergents. Another role of BAs is that of endocrine signalling molecules that activate various receptors [e.g. nuclear farnesoid X receptor (FXR) and pregnane X receptor (PXR), the G-protein-coupled receptor TGR5, etc.] to exert profound effects on hepatic lipid and glucose metabolism.[3–5]

The use of UDCA, a major component of bear bile, in the treatment of liver diseases dates back to the traditional Chinese medicine at the time of the Tang dynasty.[6,7] Today it is used for the treatment of a wide array of hepatobiliary disorders such as primary biliary cirrhosis,[8, 9] primary sclerosing cholangitis,[10] cystic fibrosis associated liver disease [11] and cholelithiasis.[12]

The prevalence and clinical application of UDCA as well as the data on participation of BAs in the pathogenesis of several liver diseases and gastrointestinal (colon) tumourigenesis provokes interest in the relationship between UDCA and cancer. Experimental evidence (*in vitro* and animal studies) suggests that UDCA may have chemopreventive actions in colorectal cancer.[13–15] UDCA has been reported to express an inhibitory effect on the induction of P-glycoprotein (responsible for multidrug resistance of tumour cells) expression and reactive oxygen species by Doxorubicin in HepG2 human hepatoma cells.[16] There are data that UDCA, particularly at low doses, may reduce the risk of advanced colorectal neoplasia (defined as colorectal cancer and/or dysplasia) in patients with primary sclerosing cholangitis with concomitant inflammatory bowel disease.[17]

It has been found in our previous investigations that UDCA decreases the viability and proliferation of cultured human and animal tumour cells in a time- and concentration- dependent manner.[18–20] To continue our research in this area, the aim of this study was to evaluate the effect of some transition metal [Zn(II), Cu(II), Ni(II)] complexes of UDCA on the viability and proliferation of cultured animal and human tumour and non-tumour cells.

## Materials and methods

### Chemicals and other materials

Dulbecco's modified Eagle's medium (DMEM) and fetal bovine serum (FBS) were purchased from Gibco-Invitrogen (UK). Dimethyl sulfoxide (DMSO), neutral red, crystal violet and trypsin were obtained from AppliChem (Germany); thiazolyl blue tetrazolium bromide (MTT) and purified agar were from Sigma–Aldrich Chemie GmbH (Germany); UDCA, Cu(NO_3_)_2_·3H_2_O, Ni(NO_3_)_2_·6H_2_O and Zn(NO_3_)_2_·6H_2_O were from Merck (Germany). All other chemicals of the highest purity commercially available were purchased from local agents and distributors. All sterile plastic and syringe filters were from Orange Scientific (Belgium).

### Synthesis of metal ursodeoxycholate complexes

Sodium ursodeoxycholate was firstly obtained by dissolving UDCA in water containing an equivalent amount of sodium hydroxide, and then the solution was evaporated to dryness on a water bath. Metal complexes were synthesized by the addition of an aqueous solution of the appropriate metal nitrate to an aqueous solution of sodium ursodeoxycholate in a 1:2 molar ratio. The resulting mixture was vigorously stirred at room temperature for 1 h. The solid complexes formed were filtered, washed several times with distilled water to eliminate unreacted metal salt and sodium ursodeoxycholate and then desiccated over P4O10. All of the solid complexes obtained were identified by elemental chemical and physico–chemical analysis (infrared, electron paramagnetic resonance and ultraviolet visible spectroscopy, magnetic measurements).[21] The compounds investigated are presented in Table 1.

**Table 1.** USDA and its metal complexes.

Compound	Abbreviation	Molecular weight (g/mol)
C_24_H_40_O_4_	UDCA	392
Zn(UDC)_2_ · 3H_2_O	Zn‒UDCA	902
Cu(UDC)_2_ · 2H_2_O	Cu‒UDCA	883
Ni(UDC)_2_ · 11H_2_O	Ni‒UDCA	1040

The compounds were dissolved in DMSO and then diluted in culture medium. The final concentration of DMSO in the stock solutions (where the concentration of the tested compound was 1 mg/mL) was 2%.

### Cell cultures and cultivation

Three groups of permanent cell lines were used as model systems in our investigations. They were established from:

1. virus-induced transplantable tumours in chicken – LSCC-SF-Mc29 (hepatoma induced by the myelocytomatosis virus Mc29) and in rat – LSR-SF-SR (sarcoma induced by Rous sarcoma virus strain Schmidt-Rupin). Both cell lines were established, characterized and maintained in the Institute of Experimental Morphology, Pathology and Anthropology with Museum – Bulgarian Academy of Sciences (IEMPAM–BAS);[22, 23];
2. human cancers of the breast (MCF-7), uterine cervix (HeLa), lung (A549) and liver (HepG2);
3. three-month-old human embryo (Lep-3).

The human cell lines were obtained from the cell culture collection of IEMPAM–BAS.

The cell cultures were grown in the DMEM medium supplemented with 5%–10% FBS, 100 U/mL penicillin and 100 μg/mL streptomycin. The cell number and viability were determined by a trypan blue dye exclusion test using a Countess® Automated Cell Counter (Invitrogen). The cultures were kept in a humidified incubator (Thermo Scientific, HEPA Class 100) at 37 °C with 5% CO_2_ in the air. For routine passages, adherent cells were detached using a mixture of 0.05% trypsin and 0.02% etylenediaminetetraacetic acid (EDTA). The cell lines were passaged two or three times per week (1:2 to 1:3 split). The experiments were performed during the exponential phase of cell growth.

### Cytotoxicity assays

The cells were seeded in 96-well flat-bottomed microplates at a concentration of 1×104 cells/well. After the cells were grown for 24 h to a sub-confluent state (∼ 60%–70%), the cells from monolayers were washed with phosphate buffered saline (PBS, pH 7.2) and covered with media modified with a solution containing different concentrations (5 μg/mL, 10 μg/mL, 50 μg/mL, 100 μg/mL and 200 μg/mL) of the compounds tested. Each solution was applied into 4–6 wells. Samples of cells grown in non-modified medium served as controls. After 24 h, 48 h and 72 h of incubation, the effect of the compounds on cell viability and proliferation was examined by an MTT test and in some cases by a neutral red uptake cytotoxicity assay (NR) and crystal violet staining (CV).

The MTT colorimetric assay of cell survival was performed as described by Mossman.[24] The method consisted of 3 h of incubation with MTT solution (5 mg MTT in 10 mL DMEM) at 37 ºC under 5% carbon dioxide and 95% air, followed by extraction with a mixture of absolute ethanol and DMSO (1:1, vol/vol) to dissolve the blue MTT formazan.

The NR assay was based on the method of Borenfreund and Puerner.[25] A medium consisting of NR (50 μg/mL, 0.1 mL) was added to each well. The plate was placed in an incubator for 3 h for the uptake of vital dye. Thereafter, the medium with NR was removed and the cells were washed with PBS (0.2 mL/well), followed by the addition of 0.1 mL 1% acetic acid solution containing 50% ethanol to extract the dye from the cells.

The CV assay was based on the method of Saotome et al.[26] After each well was washed with PBS, the cells were fixed and stained with 0.4% CV solution in methanol for 30 min.

Optical density was measured at 540 nm using an automatic microplate reader (TECAN, SunriseTM, Austria). Relative cell viability, expressed as a percentage of the untreated control (100% viability), was calculated for each concentration. Concentration–response curves were prepared and the effective concentrations of the compounds – CC50 (causing a 50% reduction of cell viability) and/or CC90 (causing a 90% reduction of cell viability) were estimated. All data points represent an average of three independent assays.

### Double staining with acridine orange (AO) and propidium iodide (PI)

The ability of compounds to induce cytopathological changes was assessed using double staining with AO and PI, according to the standard procedures.[27] The cells were grown on cover slips in 6-well plates in the presence of the compounds tested. Non-treated cells served as controls. After 24 h, 48 h and 72 h of incubation, the coverslips were removed and washed with PBS for 2 min. Equal volumes of fluorescent dyes containing AO (10 μg/mL in PBS) and PI (10 μg/mL in bi-distilled water) were added to the cells. Freshly stained cells were placed on a glass slide and examined under fluorescence microscope (Leika DM 500B, Wetzlar, Germany) within 30 min before the fluorescent colour started to fade.

### Colony-forming method

Tumour cells (103 cells/well) suspended in 0.45% purified agar in the DMEM medium containing different concentrations of the compounds examined (ranging from 5 μg/mL to 200 μg/mL) were layered in 24-well microplates. The presence/absence of colonies was scored using an inverted microscope (Carl Zeiss, Germany) over a period of 16 days. The colony inhibitory concentration (CIC) at which the compounds tested inhibit completely the ability of tumour cells to grow in semi-solid medium was determined.

### Statistical analysis

The data are presented as mean ± standard error of the mean. Statistical differences between control and treated groups were assessed using one-way analysis of variance (ANOVA) followed by the Dunnett post-hoc test and Origin 6.1TM.

## Results and discussion

In this study, we present for the first time data about the influence of ursodeoxychoic acid and its metal [Zn(II), Cu(II), Ni(II)] complexes on the viability and proliferation of animal and human tumour and non-tumour cells. The effect of the compounds on the cell viability and proliferation was studied using two trial groups performing short-term (24–72 h, with monolayer cultures) and long-term [16 days, with three-dimensional (3D) colonies] experiments, respectively.

### Short-term experiments

**Figure 1.**
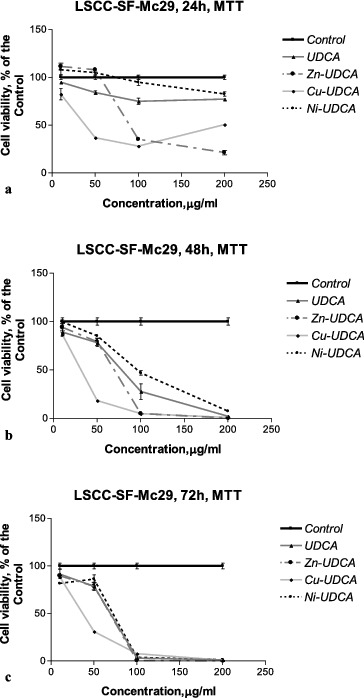
Concentration–response curves of UDCA and its metal (Zn, Cu, Ni) complexes against LSCC-SF-Mc29 chicken hepatoma cells evaluated by an MTT test after 24 h (a), 48 h (b) and 72 h (c) treatment periods.

**Figure 2.**
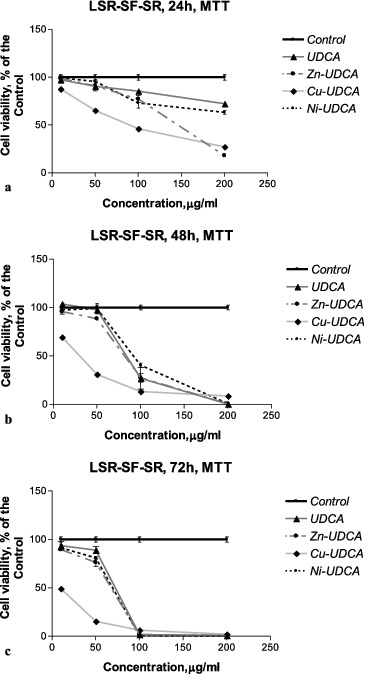
Concentration–response curves of UDCA and its metal (Zn, Cu, Ni) complexes against rat sarcoma LSR-SF-SR cells evaluated by an MTT test after 24 h (a), 48 h (b) and 72 h (c) treatment periods.

**Figure 3.**
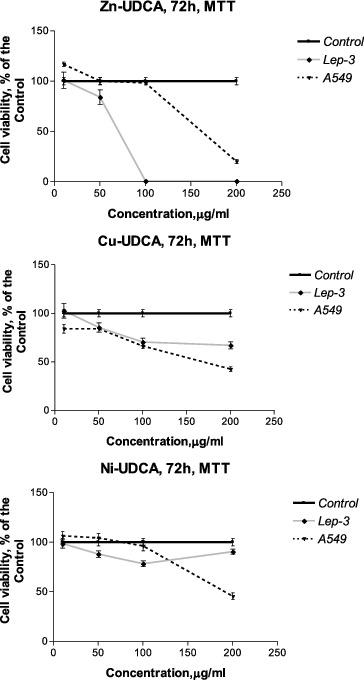
Concentration–response curves of UDCA and its metal (Zn, Cu, Ni) complexes against human A549 lung cancer and non-tumour Lep-3 cells evaluated by an MTT test after a 72 h treatment period.

**Figure 4.**
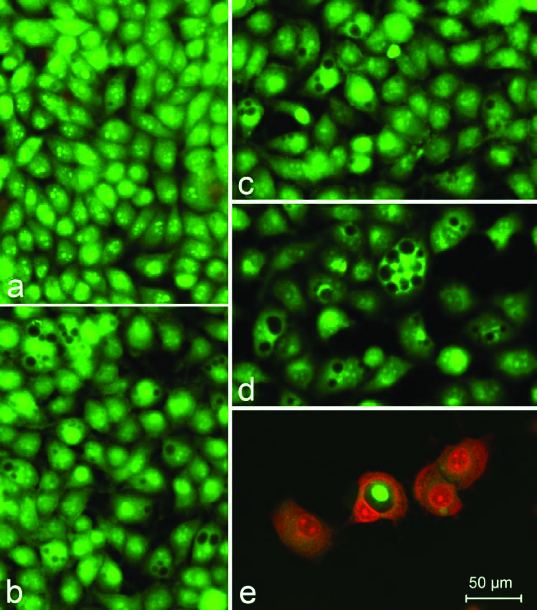
A complete monolayer of HeLa cells with a pale green nuclear fluorescence, bright yellow–green nucleoli as well as considerably more dull green fluorescence of the cytoplasm. (a) The cytoplasm includes focal perinuclear lysosomal accumulations with granular bright orange–red fluorescence. (b) HeLa cells 72 h following the treatment with 200 μg/mL UDCA. (c) 200 μg/mL Ni‒UDCA. (d) 200 μg/mL Cu‒UDCA. (e) 200 μg/mL Zn‒UDCA. Foamy vacuolation of the cytoplasm (b, c and d). Significant cell losses as well as intact and apoptotic dead cells in the (e) treatment. Acridine orange‑-propidium iodide staining.

The cytotoxic/cytostatic effects of the compounds were studied by MTT, NR and CV assays and by AO/PI staining. The results as CC50 and CC90 values (μmol/L) derived from concentration–response curves are presented in Tables 2–5. Examples of such concentration–response curves are shown in Figures 1–3. Foamy vacuolation of the cytoplasm was the main cytopathological finding (Figure 4). Significant cell losses as well as intact and apoptotic dead cells were observed after 72 h of treatment with 200 μg/mL Zn‒UDCA.

**Table 2.** Cytotoxicity (CC_50_, μmol/L) of UDCA and its metal complexes against chicken hepatoma and rat sarcoma cell lines.

			Compound			
Cell line	Method	Treatment period (h)	UDCA	Zn‒UDCA	Cu‒UDCA	Ni‒UDCA
LSCC-Mc29	MTT	24	n.d.	99.6	43.5	n.d.
		48	193.4	77.1	35.6	92.4
		72	175.5	75.7	41.7	69.1
	CV	72	n.d.	176.0	n.d.	84.0
LSR-SF-SR	MTT	24	213.2	161.9	102.2	n.d.
		48	184.2	90.5	34.1	119.8
		72	137.6	74.8	*n.d.	66.6
	NR	24	n.d.	163.5	215.1	n.d.
		48	269.8	104.4	75.0	117.6
		72	196.1	80.9	*n.d.	73.6
	CV	72	n.d.	n.d.	n.d.	n.d.

Note: MTT = thiazolyl blue tetrazolium bromide test; NR = Neutral red uptake cytotoxicity assay; CV = Crystal violet staining; n.d. = CC_50_ was not determined because at all concentrations examined the cell viability was > 50%, or *n.d.: the cell viability was < 50%.

**Table 3.** Cytotoxicity (CC_90_, μmol/L) of UDCA and its metal complexes against chicken hepatoma and rat sarcoma cell lines.

			Compound			
Cell line	Method	Treatment period (h)	UDCA	Zn‒UDCA	Cu‒UDCA	Ni‒UDCA
LSCC-Mc29	MTT	24	n.d.	n.d.	n.d.	n.d.
		48	431.3	107.0	91.7	185.8
		72	243.0	104.1	107.6	92.5
	CV	72	n.d.	221.6	n.d.	n.d.
LSR-SF-SR	MTT	24	n.d.	n.d.	n.d.	n.d.
		48	417.0	183.2	196.3	170.2
		72	242.5	104.6	148.2	90.6
	NR	24	n.d.	215.7	n.d.	n.d.
		48	461.6	200.3	n.d.	187.1
		72	246.3	104.9	110.2	93.1
	CV	72	n.d.	n.d.	n.d.	n.d.

Note: MTT = thiazolyl blue tetrazolium bromide test; NR = Neutral red uptake cytotoxicity assay; CV = Crystal violet staining; n.d. = CC_90_ was not determined because at all concentrations examined the cell viability was > 10%.

**Table 4.** Cytotoxicity (CC_50_, μM) of UDCA and its metal complexes against human tumour and non-tumour cell lines.

			Compound			
Cell line	Method	Treatment period (h)	UDCA	Zn‒UDCA	Cu‒UDCA	Ni‒UDCA
HeLa	MTT	24	n.d.	n.d.	n.d.	n.d.
		48	n.d.	n.d.	n.d.	n.d.
		72	n.d.	165.8	n.d.	n.d.
	NR	72	n.d.	147.4	n.d.	145.1
	CV	72	n.d.	152.5	n.d.	n.d.
Hep G2	MTT	24	n.d.	192.7	70.4	n.d.
		48	n.d.	135.3	172.3	171.9
		72	n.d.	179.3	n.d.	187.7
MCF-7	MTT	24	n.d.	115.4	n.d.	179.3
		48	n.d.	117.6	n.d.	88.2
A549	MTT	24	n.d.	n.d.	n.d.	n.d.
		48	n.d.	178.5	n.d.	n.d.
		72	n.d.	179.4	191.3	183.9
Lep 3	MTT	24	n.d.	78.3	n.d.	n.d.
		48	n.d.	89.3	n.d.	n.d.
		72	n.d.	77.9	n.d.	n.d.

Note: MTT = thiazolyl blue tetrazolium bromide test; NR = Neutral red uptake cytotoxicity assay; CV = Crystal violet staining; n.d. = CC_50_ was not determined because at all concentrations examined the cell viability was > 50%.

**Table 5.** Cytotoxicity (CC_90_, μM) of UDCA and its metal complexes against human tumour and non-tumour cell lines.

			Compound			
Cell line	Method	Treatment period (h)	UDCA	Zn‒UDCA	Cu‒UDCA	Ni‒UDCA
HeLa	MTT	24	n.d.	n.d.	n.d.	n.d.
		48	n.d.	n.d.	n.d.	n.d.
		72	n.d.	212.6	n.d.	n.d.
	NR	72	n.d.	205.8	n.d.	n.d.
	CV	72	n.d.	211.25	n.d.	n.d.
Hep G2	MTT	24	n.d.	n.d.	n.d.	n.d.
		48	n.d.	212.9	n.d.	n.d.
		72	n.d.	n.d.	n.d.	n.d.
MCF-7	MTT	24	n.d.	203.7	n.d.	n.d.
		48	n.d.	216.7	n.d.	n.d.
A549	MTT	24	n.d.	n.d.	n.d.	n.d.
		48	n.d.	n.d.	n.d.	n.d.
		72	n.d.	n.d.	n.d.	n.d.
Lep 3	MTT	24	n.d.	107.4	n.d.	n.d.
		48	n.d.	164.2	n.d.	n.d.
		72	n.d.	104.2	n.d.	n.d.

Note: MTT = thiazolyl blue tetrazolium bromide test; NR = Neutral red uptake cytotoxicity assay; CV = Crystal violet staining; n.d. = CC_90_ was not determined because at all concentrations examined the cell viability was > 10%.

The results obtained revealed that at the examined concentrations UDCA and its metal complexes decreased (to a varying degree) the viability and proliferation of the treated cells in a time- and concentration-dependent manner. The cytotoxic/cytostatic effects of the compounds investigated were better expressed in virus-transformed chicken hepatoma (LSCC-SF-Mc29) and rat sarcoma (LSR-SF-SR) cells (Figures 1 and 2; Tables 2 and 3). Among the tested compounds, Cu‒UDCA and Ni‒UDCA were found to be the most active cytotoxic and cytostatic agents against animal LSCC-SF-Mc29 and LSR-SF-SR cells, while Zn‒UDCA was shown to significantly decrease the viability and proliferation of human tumour cell lines. Applied independently, UDCA expressed lower cytotoxic/cytostatic activity as compared to metal complexes. The sensitivity of the non-tumour embryonic Lep-3 cells to the effects of UDCA and its metal complexes was comparable or even higher than those of the human tumour cells (Tables 4 and 5; Figure 3).

### Long-term experiments

The results on evaluation of the influence of UDCA and its metal complexes on 3D colony-formation in semi-solid medium (LSCC-SF-Mc29, LSR-SF-SR) are presented in Table 6. The data are expressed as the effective CIC (μmol/L) at which the compounds completely inhibit the tumour cell growth.

**Table 6.** Effect of UDCA and its metal complexes on colony-forming ability of tumour cells (CIC, μmol/L).

	Cell line		
Compound	LSCC-SF-Mc29	LSR-SF-SR	MCF-7
UDCA	≥ 318.4	≥ 445.8	No inhibition
Zn‒UDCA	≥ 138.6	≥ 194.0	No inhibition
Cu‒UDCA	≥ 113.3	≥ 169.9	No inhibition
Ni‒UDCA	≥ 144.2	≥ 168.3	No inhibition

According to their capacity to inhibit both the viability/proliferation and the colony-forming ability of the treated tumour cells, the compounds examined were graded in hierarchical orders that are presented in Table 7. The animal cell lines LSCC-SF-Mc29 and LSR-SF-SR were found to be most sensitive to the cytotoxic/cytostatic activities of the compounds tested. These cells contain the oncogenes *v-myc* (LSCC-SF-Mc29) and *v-src* (LSR-SF-SR) – deregulation of their cellular analogues is associated with the pathogenesis of a wide variety of human and animal cancers.[28,29] The observed cell-specific response is not surprising because the cell lines used as model system in our study differ in various characteristics such as origin (human, rat, chicken), tumour histology and etiology (spontaneous, virus-induced). Furthermore, each cancer cell line has been established from only one tumour in one patient (animal, human). Because of the tumour heterogeneity phenomenon as well as due to the changes that appear during maintenance in laboratory conditions, each (cancer) cell line is an individual system with its unique characteristics.[30]

**Table 7.** Hierarchical orders of the compounds investigated according to their cytotoxic and/or antiproliferative activities.

LSCC-SF-Mc29			
Method	According to	Treatment interval	Hierarchical order
MTT	CC_50_	48 h	Cu‒UDCA > Zn‒UDCA > Ni‒UDCA > UDCA
		72 h	Cu‒UDCA > Ni‒UDCA > Zn‒UDCA > UDCA
	CC_90_	48 h	Cu‒UDCA > Zn‒UDCA > Ni‒UDCA > UDCA
		72 h	Ni‒UDCA > Zn‒UDCA > Cu‒UDCA > UDCA
CFM	CIC	16 d	Cu‒UDCA > Zn‒UDCA > Ni‒UDCA > UDCA
LSR-SF-SR			
MTT	CC_50_	24 h	Cu‒UDCA > Zn‒UDCA > UDCA > Ni‒UDCA
		48 h	Cu‒UDCA > Zn‒UDCA > Ni‒UDCA > UDCA
		72 h	Ni‒UDCA > Zn‒UDCA > UDCA > Cu‒UDCA
	CC_90_	48 h	Ni‒UDCA > Zn‒UDCA > Cu‒UDCA > UDCA
		72 h	Ni‒UDCA > Zn‒UDCA > Cu‒UDCA > UDCA
NR	CC_50_	48 h	Cu‒UDCA > Zn‒UDCA > Ni‒UDCA > UDCA
		72 h	Ni‒UDCA > Zn‒UDCA > UDCA > Cu‒UDCA
	CC_90_	48 h	Ni‒UDCA > Zn‒UDCA > UDCA > Cu‒UDCA
		72 h	Ni‒UDCA > Zn‒UDCA > Cu‒UDCA > UDCA
CFM	CIC	16 days	Ni‒UDCA ≥ Cu‒UDCA > Zn‒UDCA > UDCA
A549			
MTT	CC_50_	72 h	Zn‒UDCA > Ni‒UDCA > Cu‒UDCA > UDCA
HepG2			
MTT	CC_50_	48 h	Zn‒UDCA > Ni‒UDCA ≥ Cu‒UDCA > UDCA

Note: MTT = thiazolyl blue tetrazolium bromide test; NR = neutral red uptake cytotoxicity assay; CFM = colony-forming method.

The non-tumour Lep-3 cells were also found to be highly sensitive to the cytotoxic/cytostatic effects of UDCA and its metal complexes. This may be explained at least partially by the embryonic origin of this line. It is known that both embryonic and cancer cells share some common properties such as rapid proliferation and low differentiation as well as expression of common antigens. Something more, tumourigenesis implies adaptation of tumour cells to an adverse environment whereas embryonic cells are usually highly sensitive to the influence of chemical substances.[31]

The existence of some variations in the antineoplastic properties of the compounds that share a very similar chemical structure could be due to at least two reasons: (1) the influence of the metal (II) ions – Zn(II), Cu(II) and Ni(II), which are known to be involved in different biological functions in living organisms, and (2) the (above-mentioned) cell-specific response.

Good correlations between the data from the short-term tests (NR and MTT, 24–72 h) in monolayer cultures and long-term colony-forming assays (16 days, 3D colonies in semi-solid medium) as well as between MTT (which reflects damage to mitochondria) and NR (indicates damage to lysosomes and Golgi apparatus) and CV (nuclear staining) methods were observed. The MTT, NR and CV assays demonstrate the ‘quick’ (24–72 h) effect of the compounds on monolayer (2D) cell cultures, whereas the colony-forming assay reveals their long-term ability to suppress the growth of tumour cell colonies (3D) in semi-solid medium. Traditional 2D cell cultures have several limitations, whereas 3D cultures more closely represent cellular function due to the increased cell-to-cell interactions and exhibiting high concordance with *in vivo* conditions.[32,33]

Added to the culture medium at concentrations similar to those in the solutions of the metal complexes tested, DMSO did not lead to noticeable changes in the survival of the treated cells. This fact is not surprising, since in the stock solution (where the concentration of the test substance is 1 mg/mL), the DMSO content is 2% and in the solutions tested (where the concentration of the test substance is 5 μg/mL to 200 μg/mL) the share of DMSO progressively decreases (from 0.4% to 0.01%).

The data obtained deserve interest because: (1) secondary BAs have been proved to express both detrimental and cell-protective effects [2]; (2) UDCA is currently used in the treatment of various diseases and has been accepted as a therapeutic standard in some of them (such as primary biliary cirrhosis) [34, 35]; and (3) the examined compounds are the complexes of UDCA with metals Zn(II), Cu(II), Ni(II) that are widely distributed in living organisms. These facts as well as the suspected relationship between BAs and cancer give rise to the need to conduct such studies, since the effect of metal complexes with UDCA on tumour/non-tumour cell viability and proliferation has not been clarified yet.

## Conclusions

To the best of our knowledge, this study for the first time demonstrates the ability of metal [Zn(II), Cu(II), Ni(II)] complexes with UDCA to express cytotoxic and antiproliferative properties against animal and human tumour cell lines of various origins, being more active in retrovirus-transformed chicken hepatoma (LSCC-SF-Mc29) and rat sarcoma (LSR-SF-SR) cells. The knowledge concerning the relationship between chemical structure of such compounds and their biological activities will facilitate the design of drugs with increased anticancer potential and improved biocompatibility.
